# The PPARgamma-selective ligand BRL-49653 differentially regulates the fate choices of rat calvaria versus rat bone marrow stromal cell populations

**DOI:** 10.1186/1471-213X-8-71

**Published:** 2008-07-14

**Authors:** Takuro Hasegawa, Kiyoshi Oizumi, Yuji Yoshiko, Kazuo Tanne, Norihiko Maeda, Jane E Aubin

**Affiliations:** 1 Department of Orthodontics, Hiroshima University Graduate School of Biomedical Sciences, 1-2-3 Kasumi, Minami-ku, Hiroshima 734-8553, Japan; 2 Department of Molecular Genetics, University of Toronto, Toronto, Ontario M5S1A8, Canada; 3 Department of Oral Growth and Developmental Biology, Hiroshima University Graduate School of Biomedical Sciences, 1-2-3 Kasumi, Minami-ku, Hiroshima 734-8553, Japan; 4 Biological Research Laboratories III, Daiichi Sankyo Co., Ltd. 1-16-13 Kitakasai, Edgawa-ku, Tokyo 134-8630, Japan

## Abstract

**Background:**

Osteoblasts and adipocytes are derived from a common mesenchymal progenitor and an inverse relationship between expression of the two lineages is seen with certain experimental manipulations and in certain diseases, *i.e*., osteoporosis, but the cellular pathway(s) and developmental stages underlying the inverse relationship is still under active investigation. To determine which precursor mesenchymal cell types can differentiate into adipocytes, we compared the effects of BRL-49653 (BRL), a selective ligand for peroxisome proliferators-activated receptor (PPAR)γ, a master transcription factor of adipogenesis, on osteo/adipogeneis in two different osteoblast culture models: the rat bone marrow (RBM) versus the fetal rat calvaria (RC) cell system.

**Results:**

BRL increased the number of adipocytes and corresponding marker expression, such as lipoprotein lipase, fatty acid-binding protein (aP2), and adipsin, in both culture models, but affected osteoblastogenesis only in RBM cultures, where a reciprocal decrease in bone nodule formation and osteoblast markers, *e.g*., osteopontin, alkaline phosphatase (ALP), bone sialoprotein, and osteocalcin was seen, and not in RC cell cultures. Even though adipocytes were histologically undetectable in RC cultures not treated with BRL, RC cells expressed PPAR and CCAAT/enhancer binding protein (C/EBP) mRNAs throughout osteoblast development and their expression was increased by BRL. Some single cell-derived BRL-treated osteogenic RC colonies were stained not only with ALP/von Kossa but also with oil red O and co-expressed the mature adipocyte marker adipsin and the mature osteoblast marker OCN, as well as PPAR and C/EBP mRNAs.

**Conclusion:**

The data show that there are clear differences in the capacity of BRL to alter the fate choices of precursor cells in stromal (RBM) versus calvarial (RC) cell populations and that recruitment of adipocytes can occur from multiple precursor cell pools (committed preadipocyte pool, multi-/bipotential osteo-adipoprogenitor pool and conversion of osteoprogenitor cells or osteoblasts into adipocytes (transdifferentiation or plasticity)). They also show that mechanisms beyond activation of PPARγ by its ligand are required for changing the fate of committed osteoprogenitor cells and/or osteoblasts into adipocytes.

## Background

Osteoblasts and adipocytes derive from a common mesenchymal progenitor and appear to display plasticity between the phenotypes and an inverse relationship between expression of the two lineages under certain pathological and several experimental conditions [1]. For example, a reciprocal relationship between marrow adipocyte content and bone mass has been reported in osteoporosis [2]. Studies on the senescence-accelerated (SAMP6) [3] and aging mouse [4] models also suggest that osteoblastogenesis is decreased concomitant with an increase in the number of marrow adipocytes.

As summarized previously [5], however, it can often be difficult to interpret the developmental and cellular basis of the inverse relationship between osteoblasts and adipocytes, when both bi-/multipotential progenitors reside in the same populations as restricted monopotential progenitors that may display plasticity or transdifferentiation capacity. Data from different approaches confirm multiple possible cellular events underlying transitions between the two lineages (see, *e.g*., [6,7]). Thus, the number of adipocytes in bone marrow stromal or bone-derived populations may reflect the frequency of committed adipocyte precursors (pathway 2), the conversion of osteoprogenitor cells and/or osteoblasts into adipocytes (pathway 3), and changes to the balance in commitment choices of mesenchymal stem cells (pathway 1). Specific treatments and different culture conditions including different cell densities may dependently or independently affect any or all of these lineage choices.

Adipocyte differentiation is under the control of peroxisome proliferator-activated receptors (PPARs), members of the nuclear receptor superfamily, in concert with members of the CCAAT/enhancer-binding protein (C/EBP) family of basic leucine zipper nuclear transcription factors [8]. The analyses of homo- and heterozygous PPARγ-deficient mice and ES cells have suggested that PPARγ may positively and negatively determine the fate of osteo-adipocyte precursors respectively at least during early differentiation events [7]. The thiazolidinedione antidiabetic agents, which are PPARγ-selective ligands, induce adipogenesis in a variety of culture models including mesenchymal stem/stromal cells and cell lines (see, *e.g*., [9-12]). However, different ligands of the thiazolidinedione class with different capacities for PPARγ activation appear to differentially modulate adipogenesis versus osteoblastogenesis in the mouse model [12]. Taken together, these reports suggest that PPARγ-selective ligands may induce adipogenesis not only in mesenchymal stem or multipotential progenitor cells (pathway 1), but also in osteoprogenitors and/or osteoblasts (pathway 3).

Three members of the C/EBP family (C/EBPα, β, and δ) have also been implicated in adipocyte differentiation [8]. Analyses of the differentiation program in adipocytic cell lines and genetically altered mice have shown that C/EBP and PPAR work sequentially and cooperatively to stimulate the molecular events required for adipogenesis. C/EBPβ and δ also activate osteocalcin gene transcription and synergize with runt-related transcription factor 2 (Runx2), a master regulator for osteoblastogenesis, at the C/EBP element to regulate bone-specific expression [13]. To elucidate the contribution of recruitment from a committed osteoblast precursor pool (pathway 3) versus multipotential progenitor pool (pathway 1) to adipogenesis induced by the PPARγ-selective ligand BRL-49653 (BRL), we compared primary cultures of fetal rat calvaria (RC) cells (in which osteoblasts derive mainly from committed osteoprogenitors, a model of pathway 3, and in which committed preadipocytes also reside, a model of pathway 2) with rat bone marrow (RBM) cultures (representing a model of pathway 1) [1].

## Results

**Figure 1:**
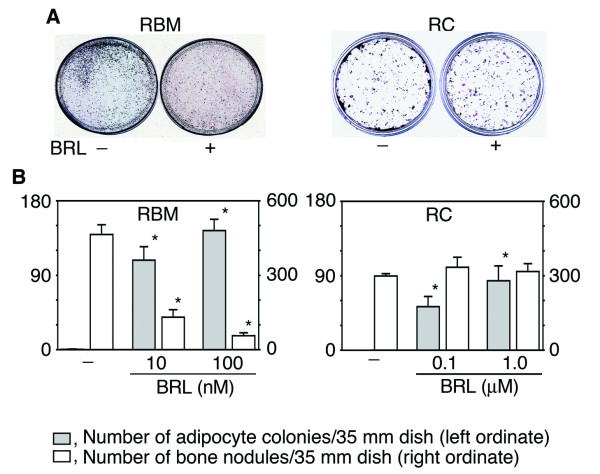
**BRL exerts different effects on bone nodule and adipocyte colony formation in RBM versus RC cell cultures.** Cells were fixed at day 17 (RBM) or day 18 (RC). A, Oil red O/von Kossa staining. B, The number of adipocyte colonies and bone nodules. BRL at 10 and 100 nM and 0.1 and 1.0 μM were used for RBM and RC cells, respectively. Data are mean ± SD of triplicate samples; results are representative of a minimum of three independent experiments. *p< 0.05 vs. vehicle.

**Figure 2:**
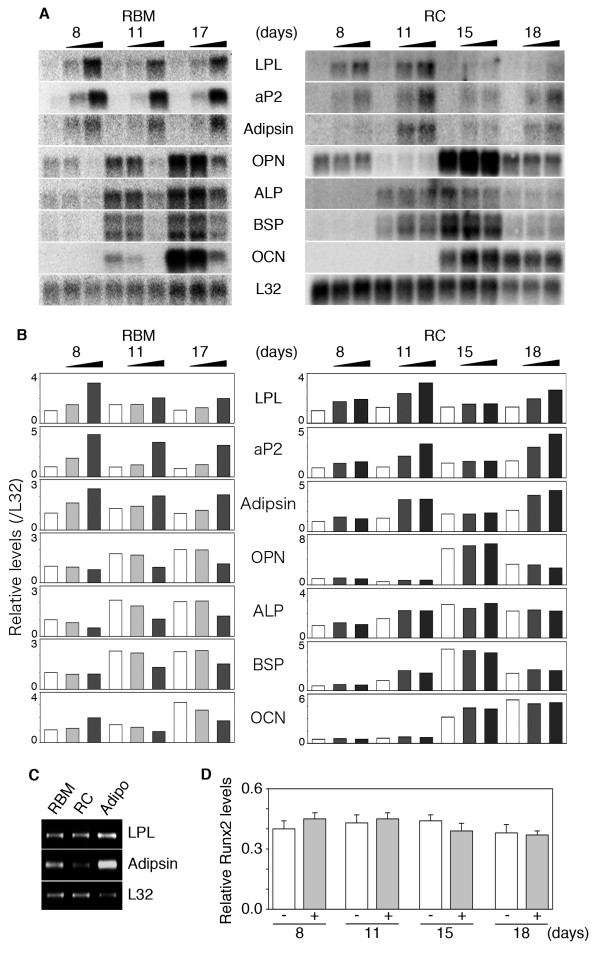
**Expression of adipocyte and osteoblast markers is reciprocally affected by BRL in RBM but not in RC cells.** A Northern blot analysis of adipocyte and osteoblast markers; B Quantitative analysis by densitomtery. Adipocyte markers, LPL, aP2, and adipsin. Osteoblast markers, OPN, ALP, BSP, and OCN. RBM and RC cells were treated with BRL at 10 and 100 nM and 0.1 and 1.0 μM (indicated by increasing bar size), respectively, as shown in Fig. 1. Total RNA was harvested at appropriate days as indicated. C, RT-PCR analysis of LPL, adipsin and L32 in RC cell and RBM treated with BRL and in brown adipose tissue as a positive control. Samples of RC and RBM (in the presence of BRL at 1 μM and 100 nM, respectively) are the same as those in A. Adipo, brown adipose tissue from 21-day fetal rats. D, Real-time RT-PCR of Runx2 in RC cells treated with (+) and without (-) 1 μM BRL. Samples are the same as those in A. L32, internal control. Data are mean ± SD of triplicate samples; results are representative of a minimum of three independent experiments.

The temporal sequence of osteoblast development in both the RBM and RC cell culture models has been well-described, and data support the concept that the models reflect a preponderance of multi/bipotential progenitors in the RBM system versus a preponderance of osteoprogenitors in the RC cell system [1,14]. To determine whether there is a difference in the effects of BRL on osteoblastogenesis between these two models, we first assessed the number of bone nodules and adipocyte colonies in RBM versus RC cell cultures treated with BRL (Fig. 1A and 1B). BRL increased the number of adipocyte colonies formed in both culture models, while it markedly decreased bone nodule formation in RBM but had no detectable effect on bone nodule formation in RC cells even at 10-fold higher concentrations (1.0 μM) than used for RBM cultures. During bone nodule formation in both RBM and RC cell cultures, the expected sequential marked upregulation of osteoblast markers was seen, while adipocyte markers remained relatively low and stable (Fig. 2A,B). In agreement with its effects on bone nodule/adipocyte colony formation, BRL increased adipocyte marker (lipoprotein lipase (LPL), fatty acid-binding protein (aP2), and adipsin) and decreased osteoblast marker (osteopontin (OPN), alkaline phosphatase (ALP), bone sialoprotein (BSP), and osteocalcin (OCN)) mRNA levels throughout the time course in RBM cultures (Fig. 2A,B). Also consistent with the BRL-induced increase in adipocyte colony number in RC cultures, expression of adipocyte markers was increased by BRL treatment of RC cells at all time points, but especially days 11 and 18 (Fig. 2A,B). Compared to the fetal adipose tissue control (Fig. 2C), expression levels of adipocyte markers are lower in the BRL-treated RC and RBM cell cultures, but consistent with the frequency of adipocytic versus non-adipocytic cells in each case (adipose tissue>RBM>RC; see also Fig. 1) and rosiglitazone effects *in vivo*[15]. On the other hand and in parallel with the lack of effect of BRL on the number of bone nodules formed (Fig. 1), osteoblast marker mRNA levels were either unchanged or changed only slightly by BRL at all doses tested, *i.e*., ALP and BSP levels were slightly upregulated at day 11 (Fig. 2A,B). Notably, expression of the mRNA for osteoblast master regulator gene, Runx2, was not affected by BRL treatment at any time in RC cell cultures (Fig. 2D).

**Figure 3:**
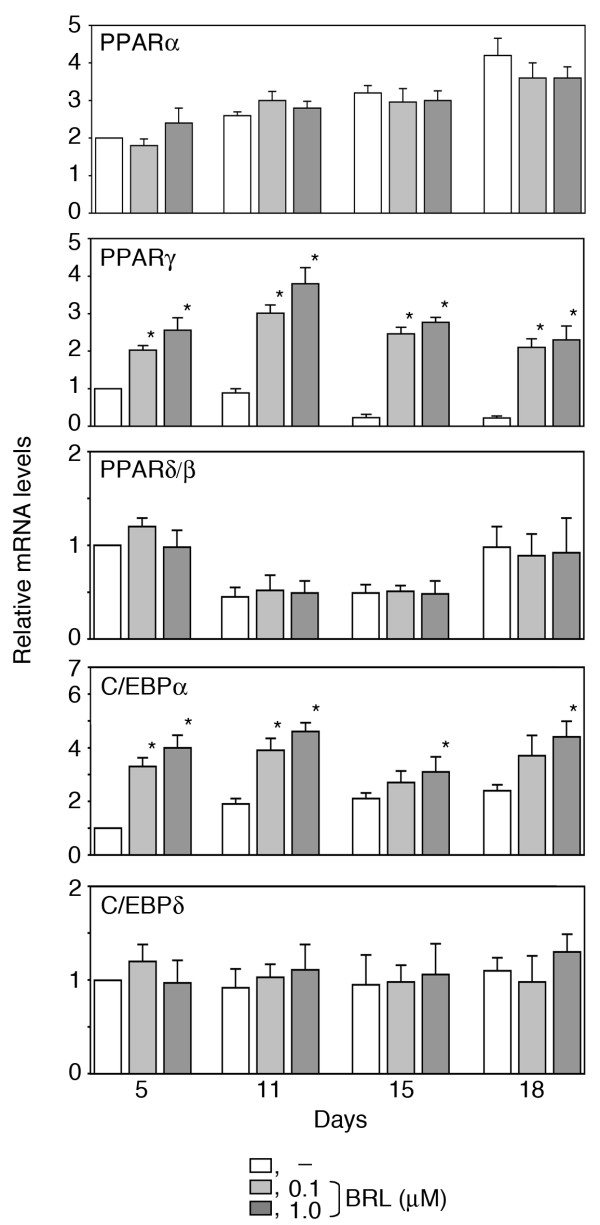
**Expression profiling of PPARs and C/EBPs in the presence or absence of BRL during osteoblast development in RC cell cultures.** Total RNA was collected as described in Fig. 2. Real-time RT-PCR was carried out by using specific primer sets for PPARα,γ, δ/β, C/EBPα, δ and L32. The ratio was calculated against the values of vehicle at day 5 that was set at 1.0. Data are mean ± SD of triplicate samples; results are representative of three independent experiments. *p< 0.05 vs. time-matched values of vehicle.

To address whether the lack of suppression of osteoblastogenesis by BRL in RC cell cultures was due to the absence of its receptor, PPARγ, or other transcription factors involved in the adipogenesis cascade, real-time PCR of the PPAR and C/EBP family members was performed on RNA from RC cells throughout the differentiation time course (Fig. 3). Amongst molecules examined, C/EBPβ was found only after robust amplification (41 cycles in regular RT-PCR) and, even then, was not seen consistently in RC samples (data not shown). On the other hand, PPAR mRNAs as well as C/EBPα mRNAs were detected over the time course of osteoblast development; PPARγ expression decreased, while the expression of PPARα and C/EBPα gradually increased, and PPARδ/β and C/EBPδ remained unchanged. Amongst these transcription factors, PPARγ and C/EBPα expression was significantly increased in BRL-treated RC cells at all times tested (Fig. 3), raising the possibility that BRL may convert at least some osteogenic cells or their precursors towards an adipogenic phenotype.

**Figure 4:**
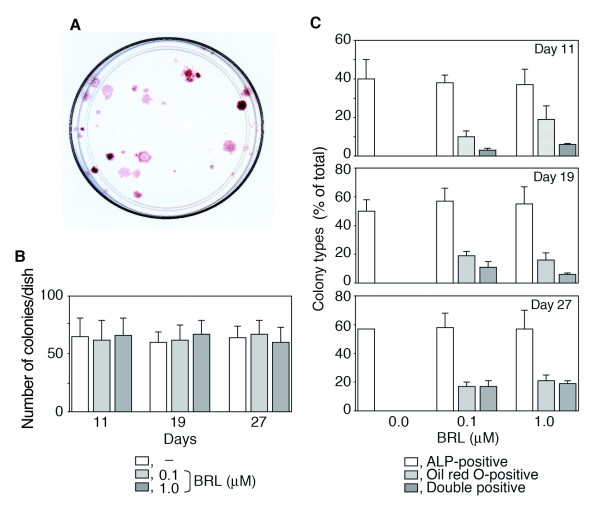
**BRL increases adipocyte colonies without affecting osteoblastic colony formation.** RC cells plated at 500 cells/100 mm dish were fixed at the days indicated and stained for adipogenesis (oil red O) and osteogenesis (ALP). A, Typical single cell colonies stained with ALP/von Kossa (day 27). B, Total number of CFU-F per 35-mm dish in the presence and absence of BRL at the concentrations indicated (day 27). C, The proportion of total colonies that are oil red O^+ ^and/or ALP^+ ^colonies in RC cells treated with and without BRL. Note that the proportion of the colonies showing a double (osteoblast plus adipocyte) phenotype increased with time. Data are mean ± SD of triplicate samples; results are representative of three independent experiments. *p< 0.05 vs. vehicle.

Because RC cell cultures comprise a heterogeneous mix of osteoprogenitor cell types at multiple differentiation stages [16] as well as a small number of fibroblastic cells, adipocyte precursors [17] and a small multipotential side population [18], we next used a more discriminating assay than total population analysis to address the question of whether osteogenic cells can (trans)differentiate into adipocytes when treated with BRL. We therefore plated RC cells at very low density and analysed single cell-derived isolated colonies (in this experiment, a total of 732 colonies in 4 independent dishes (194 colonies at day 11, 249 colonies at day 19 and 289 colonies at day 27); Fig. 4A shows a representative 100 mm dish plated at low density for the colony forming unit (CFU) assay). We confirmed that BRL treatment had no detectable effect on the growth rate or total number of CFUs (Fig. 4B), ruling out a generalized mitogenic or toxic effect of BRL in the RC cell population. When we quantified the phenotypes of individual colony types present, we found that BRL had no significant effect on the number of colonies with an osteoblastic phenotype (CFU-ALP), but induced oil red O-single positive (adipocytic) as well as ALP/oil red O-double positive (osteo–adipocytic) colonies. Note especially that many of the adipocyte colonies also contained ALP-positive cells (Fig. 4C). The percentage of such double-stained colonies increased over the time course of the experiment, such that by the end of the experiment (day 27), a large number of oil red O-positive colonies induced by BRL also had ALP-positive cells, suggesting that BRL induced expression of both the osteoblast and adipocyte developmental programs in some RC progenitor cells.

**Figure 5:**
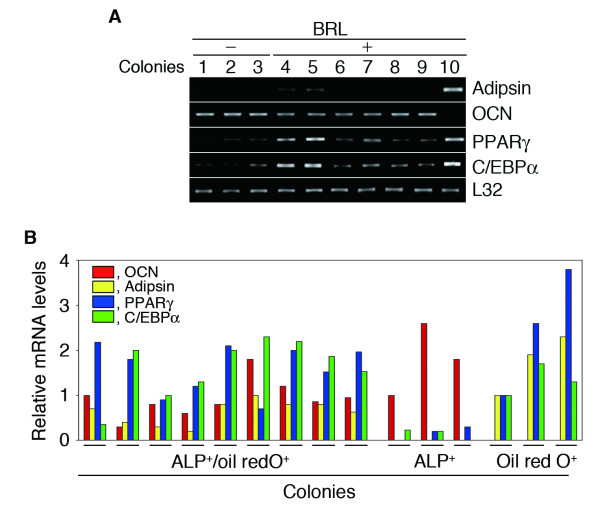
**Gene expression profiling of osteoblast and adipocyte markers in single cell-derived colonies.** Total RNA was obtained at day 33 from each randomly selected colony as shown in Fig. 4. A, A representative agarose gel with amplimers from RT-PCR for adipsin, BSP, OCN, PPARγ, and C/EBPα. L32, internal control. Samples shown in lanes 1–3 and lanes 4–9 were collected from randomly selected ALP^+ ^colonies in vehicle and BRL-treated cultures, respectively. One adipocyte colony (lane10) was also collected from a BRL-treated culture. B, Expression profiling of osteo/adipocyte marker mRNAs in ALP^+^/oil red O^+ ^colonies in cultures treated with or without BRL. Data shown are from 9 ALP^+^/oil red O^+ ^colonies; also shown for comparison are three ALP^+ ^and three oil red O^+ ^colonies. Data are representative of three independent experiments.

We next assessed adipocyte and osteoblast marker expression at day 33 in individual colonies with definitive osteoblast (cuboidal ALP-positive, ALP^+ ^cells) or definitive adipocyte (pleomorphic cells with patent lipid droplets) or mixed (containing both cuboidal ALP^+ ^and lipid-containing cells) phenotypes as defined morphologically; BRL induced a significant shift in colony type from single ALP^+ ^colonies to double ALP^+^/Oil red O^+ ^colonies (*p *< 0.01). A representative agarose gel with amplimers obtained by semiquantitative RT-PCR of RNA from 10 randomly selected colonies is shown in Fig. 5A: 3 ALP^+ ^colonies (lanes 1–3) from vehicle-treated culture dishes, and 6 ALP^+ ^colonies (lanes 4–9) and 1 colony with typical adipocyte morphology (oil red O^+ ^lipid droplets; lane 10) from BRL-treated culture dishes (Fig. 5A). Representative results from quantification of osteo/adipocyte marker mRNAs in 9 double ALP^+^/oil red O^+ ^colonies in comparison to three ALP^+ ^and three oil red O^+ ^colonies are shown in Fig. 5B. A summary of results from 68 randomly selected colonies (9 of which were also oil red O^+^) and 25 ALP^+ ^colonies from cultures grown in the presence or absence of BRL respectively, and 3 oil red O^+ ^colonies from BRL-treated cultures) is shown in Table 1. Of the 25 ALP^+ ^colonies formed in the absence of BRL, 22 colonies expressed OCN, but none of them expressed adipsin; 10 of these also expressed PPARγ or C/EBPα and 7 colonies expressed both (PPARγ^+^/C/EBPα^+^). The 3 adipocyte (Oil red O^+ ^colonies from the BRL-treated cultures expressed adipsin, PPARγ and C/EBPα, but had no detectable OCN expression. Of 40 ALP^+ ^colonies formed in the presence of BRL, 27 colonies expressed OCN, and 9 colonies co-expressed OCN, adipsin and PPARγ and C/EBPα (Fig. 5B; Table 1). BRL induced a significant shift in colony types from ones expressing only one of PPARγ or C/EBPα to ones expressing both factors (*p *< 0.05), and from ones expressing OCN alone to those expressing both OCN and adipsin, as well as PPARγ and C/EBPα (*p *< 0.01). Taken together with the morphological assessment of colony types, our results suggest that at least some osteoprogenitor cells are induced by BRL to upregulate an adipogenic differentiation program.

**Table 1:** The effect of BRL on osteo/adipogenic differentiation outcomes in RC single cell-derived colonies

		Colony Number				
		
		BRL				Total
			
		-		+		
	(Total)	25	%	43	%	68
CFU	ALP^+^	25	100	31	72	56
	Oil red O^+^	0	0	3	7	3
	ALP^+^/Oil red O^+^	0	0	9**	21	9
	ALP^-^/Oil red O^-^	0	0	0	0	0

	OCN^+^	22	88	27	63	49
	Adipsin^+^	0	0	3	7	0
	OCN^+^/Adipsin^+^	0	0	9**	21	9
	OCN^-^/Adipsin^-^	3	12	4	9	7
Gene expression	PPARγ^+^	4	16	3	7	7
	C/EBPα^+^	6	24	5	12	11
	PPAR γ^+^/C/EBPα^+^	7	28	31*	72	35
	PPARγ^-^/C/EBPα^-^	8	32	4	9	12
	OCN^+^/Adipsin^+^/PPARγ^+^/C/EBPα^+^	0	0	9**	21	9

** *p *< 0.01 vs. no BRL; *p < 0.05 vs. no BRL.

## Discussion

**Figure 6:**
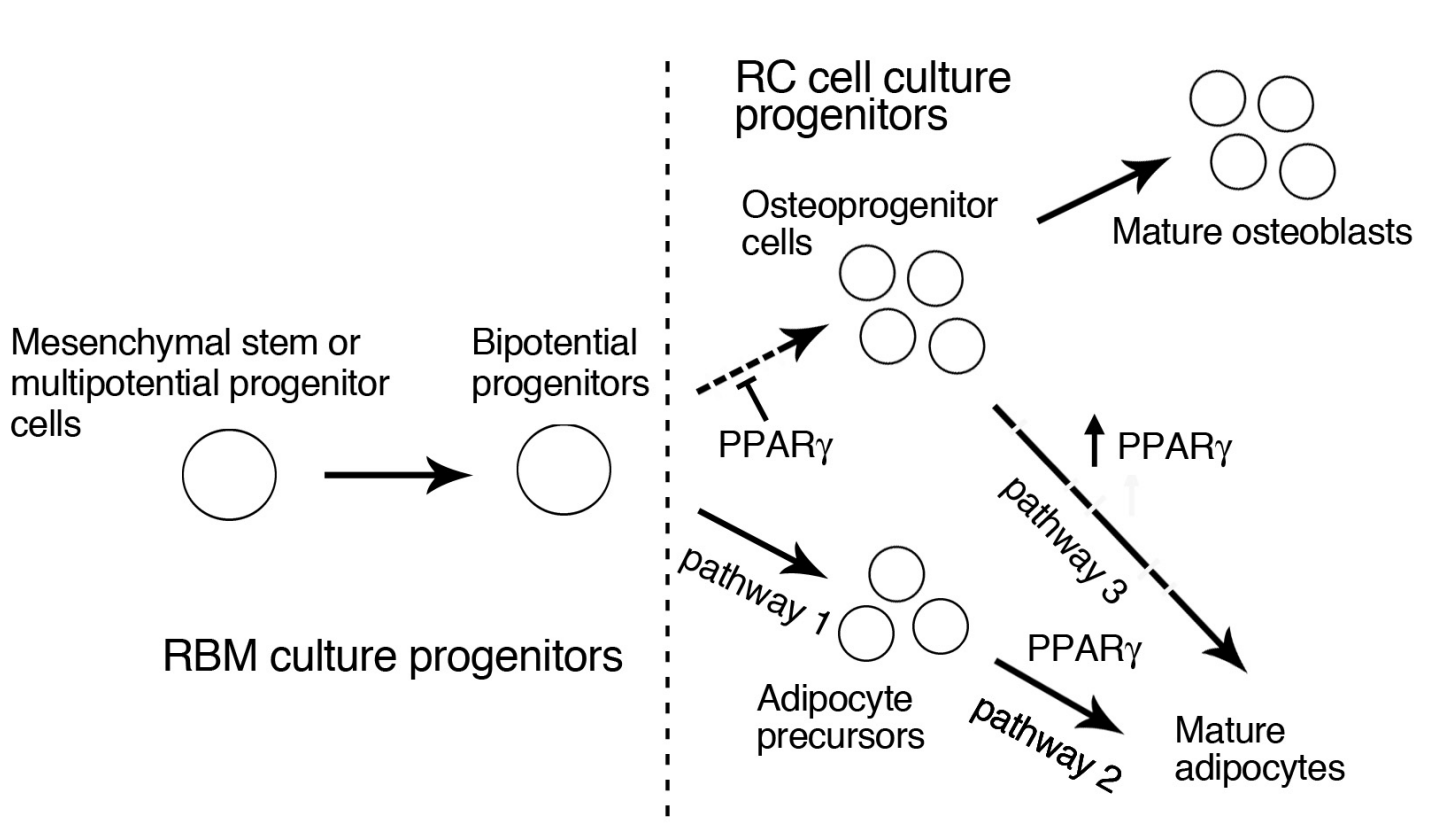
**Possible cellular origin of adipocytes induced by BRL.** The number of adipocytes may be influenced by a number of recruitment pathways, including from commitment of mesenchymal stem cells or bipotential progenitors to the preadipocyte pool (pathway 1), from maturation of committed adipocyte precursors (pathway 2) and the conversion of committed osteoprogenitor cells and/or osteoblasts into adipocytes via transdifferentiation or plasticity (pathway 3). BRL directs mesenchymal stem or multipotential progenitor cells in RBM to adipocytes at the expense of osteoblast differentiation. A portion of osteoprogenitor cells in RC cells may be able to express both osteoblast and adipocyte phenotypes in response to BRL. A portion of committed adipocyte precursors in RC populations also responds to BRL with an increase in adipogenesis.

Treatment of mouse or rat (RBM) stromal cell cultures with BRL-49653 (BRL), a selective ligand for PPARγ, elicits reciprocal effects on adipogenesis and osteoblastogenesis, stimulating the former and inhibiting the latter, as described [1-4]. In contrast, the ligand does not alter the functional fate or endpoint of committed osteoprogenitors resident in RC populations, *i.e*., formation of mineralized bone colonies is not altered, even though adipogenesis is induced in this population. On the other hand, some single cell-derived osteoblastic colonies in BRL-treated but not untreated RC populations co-express markers of both mature osteoblast and adipocyte. These results suggest a clear difference in the progenitor status between the two populations and support the conclusion that BRL may show capacity to recruit adipocytes from multiple precursor pools (Fig. 6).

It is worth considering that because RBM (from mesoderm) and RC cells (from a mixture of neuroectoderm and mesoderm) are embryologically distinct, the regulation of fate selection by progenitors in the stromal versus calvarial populations may be different. For example, some hormones and growth factors regulate intramembranous or periosteal bone formation (calvaria or periosteal growth of other bones) differently than endosteal bone formation (stromal cell-derived trabeculae and endosteal growth) (*e.g*., parathyroid hormone effects; [19]). Thus, RC and RBM cells may be subject to differential regulation by activation of PPARγ (BRL), while responding similarly to other factors, *e.g*., dexamethasone [1,5,14] and endogenous glucocorticoids as evidenced by 11β-hydroxysteroid dehydrogenase type 2-overexpressing mice driven by α1(I)-collagen promoter [20].

Bone marrow stromal cell populations, often referred to as mesenchymal stem cells, are capable of undergoing differentiation along multiple mesenchymal lineages, but are heterogeneous in the capacity of individual colonies (CFU-F) to express multilineage versus more restricted capacity (see, *e.g*., [21], and discussion in [1]). However, the frequency of multipotential progenitors in stromal populations appears quite high, *e.g*., ~30% of single cell-derived colonies in human stroma [22] and >90% in a recently-described alternative isolation/enrichment method [21]. As described above, RC cell populations also contain a mixture of cell lineages and types, *e.g*., osteoprogenitors and osteoblasts at different differentiation stages, fibroblastic cells, adipocyte precursors [17], as well as a multipotential side population [18]. However, the frequency of functionally multipotential precursor or stem cells, *e.g*., the RCJ3.1, clonally-derived multipotential cell line [23], bipotential adipo-osteoprogenitors [24] or SP cells [18] appears to be very low in RC populations. Additionally, RC populations contain preadipocytes [25,26] (pathway 2; Fig. 6) and possibly circulating progenitor cells from bone marrow [27]. Our data support the view that BRL induces differentiation/maturation of adipocytes mainly from a committed preadipocyte pool in RC populations. However, our data on single cell colonies suggest that a subpopulation of committed osteoprogenitors or relatively mature osteoblasts is also induced to switch on the adipogenic pathway (pathway 3) when PPARγ is activated, as we also recently proposed with leukemia inhibitory factor treatments [26]. The expression of PPARγ and/or C/EBPs in some osteogenic cells in our models and MC3T3-E1 cells [28] may also predispose them to the pathway 3. It is also possible that at least some of the osteo-adipogenic cells in BRL-treated RC populations represent recruitment from multipotential or bipotential progenitor pools equivalent to those in stromal cell populations; the low frequency of such cells in RC populations would not be expected to markedly alter overall osteoblast or adipocyte colony numbers (Fig. 4, 5A, and see [14]), although we cannot discount the possibility that activation of PPARγ dramatically changes their frequency.

In BRL-treated single cell colonies, mineralized colonies with and without adipsin expression (Fig. 5B) correlated with high and low levels of PPARγ respectively, which is consistent with the hypothesis that RC cell populations comprise two kinds or differentiation stages of progenitor or osteoblastic cells as we described previously with respect to glucocorticoid regulation [29,30], *i.e*., a subpopulation responsive to the PPARγ selective ligand and a non-responsive subpopulation, with the former capable of expressing both the adipocyte and osteoblast programs and the latter expressing only the osteoblast program. Our data are also consistent with the view that activation of PPARγ alone by its ligand is not sufficient to induce complete conversion of osteoprogenitor cells into adipocytes. This view is consistent with an earlier report in which the PPARγ selective ligand was unable to increase PPARγ expression and cause adipocyte differentiation in human adult trabecular-derived bone cells, although these cells were able to undergo adipogenesis in the presence of isobutylmethylxanthine (IBMX) plus dexamethasone with concomitant increase in expression of PPARγ [31]. Treatment of MC3T3-E1 cells retrovirally overexpressing PPARγ with insulin, dexamethasone and IBMX increases the adipogenic capacity, which is further enhanced when C/EBPα is co-overexpressed [32]. Together these data suggest that mechanisms beyond activation of PPARγ by its ligand (BRL) are required for changing the fate of committed osteoprogenitor cells and/or osteoblasts into adipocytes.

BRL is a potent PPARγ-selective ligand but it is also known to increase PPARγ expression [33], as it did in RC cells, along with C/EBPα. These two transcription factors display interactive regulatory roles and cooperate to promote adipocyte differentiation [34]. The role of these two factors, or other members of the families in osteoblasts has been investigated recently (see *e.g*., [13]). Wnt/β-catenin signaling suppresses C/EBPα and PPARγ, which shifts mesenchymal cell fate toward osteoblastogenesis at the expense of adipogenesis [35,36]. The lack of coordinate expression of PPAPγ and C/EBPα during osteoblast differentiation in our models suggests that they may not cooperate in osteoblast differentiation as they do in adipocyte differentiation.

## Conclusion

The present study showed clear differences in the capacity of the PPARγ-selective ligand BRL-49653 to alter the fate choices of precursor cells in stromal versus calvarial cell populations and that recruitment of adipocytes can occur from multiple precursor cell pools (committed preadipocyte pool, multi-/bipotential osteo-adipoprogenitor pool and conversion of osteoprogenitor cells or osteoblasts into adipocytes (transdifferentiation or plasticity)). They also show that mechanisms beyond activation of PPARγ by its ligand are required for changing the fate of committed osteoprogenitor cells and/or osteoblasts into adipocytes.

## Methods

### Cell cultures

Animal use and procedures were approved by the University of Toronto Animal Care Committee and Research Facilities for Laboratory Animal Science, Natural Science Center for Basic Research and Development, Hiroshima University.

### RBM stromal cell cultures

Bone marrow stromal cells from the femora of young adult male Wistar rats (110–130 g) were cultured essentially as described [37]. Briefly, femora were dissected and immersed α-MEM with antibiotics. After removal of the epiphysis, the marrow was collected by flushing MEM, supplemented with antibiotics and 10% fetal calf serum (FCS), through the shafts with a syringe. The resulting cell suspension was plated into a T75-tissue culture flask and incubated in the same medium supplemented additionally with ascorbic acid (50 μg/ml) and dex (10 nM) (differentiation medium) for a week at 37°C in a humidified atmosphere of 95% air and 5% CO_2_. Cells were then harvested with trypsin and collagenase and subcultured at 0.2 × 10^4 ^cells/cm^2 ^in differentiation medium; medium was changed every 2–3 days until bone nodules were observed. Cells were treated chronically with or without BRL (10–100 nM). To promote mineralization, 10 mM β-glycerophosphate was added for the last 5 days.

### RC Cell Cultures

Cells were enzymatically isolated from calvariae of 21-d Wistar rat fetuses by sequential digestion with collagenase as described [38]. Cells obtained from the last four of five digestion steps were grown in α-MEM containing 10% FCS and antibiotics. After 24 h, cells were collected by trypsinization, and cultured at the same cell density as RBM in the presence or absence of BRL (0.1–1 μM) in differentiation medium as above but without dex. To obtain single cell colonies, RC cells were also cultured at very low density (500 cells/100 mm dish) in differentiation medium [16].

### Northern Blots

Total RNA was harvested at appropriate culture time points with TRIzol reagent (Invitrogen, Carlsbad, CA) according to the manufacturer's instructions. Twenty micrograms of total RNA were electrophoresed on 1% agarose-17% formaldehyde gels and transferred onto positively charged nylon membranes (Hybond-N^+^, GE Healthcare, Buckinghamshire, UK). The membranes were cross-linked, prehybridized and hybridized with specific probes as described (see below, and [39]). After washing, the membranes were exposed to X-ray film at -80°C for various times. cDNAs for rat BSP (pBSP1), OCN (pOC9), ALP and OPN were described previously [37]. The aP2 and adipsin cDNAs were cloned from BRL-treated mouse bone marrow cells, using specific primers designed with Primer Picking (Primer 3). The primer sequences were as follows: aP2, 5'-ATA GCA CCC TCC TGT GCT G-3' and 5'-CCA GCC TCT TCC TTT GCT C-3'; adipsin, 5'-TGT ACT TCG TGG CTC TGG TG-3' and 5'-ATC CGG TAG GAT GAC ACT CG-3'. A mouse LPL cDNA was purchased from the American Type Culture Collection (63117; Rockville, MD). All probes were labeled with [α^32^P]dCTP using a Multiprime DNA labelling system (GE Healthcare). L32 was used as internal control.

### Real-time and semiquantitative RT-PCR

cDNA was synthesized from 2 μg or less (100–400 ng from single cell colonies) of total RNA isolated from cells and tissues as above, using Superscript II (Invitrogen) or RevatraAce (Toyobo, Osaka, Japan). The sequence of PCR primers were designed using Primer 3; PPARα, 5'-CGA CAA GTG TGA TCG AAG CTG CAA G-3' and 5'-GTT GAA GTT CTT CAG GTA GGC TTC-3'; PPARδ/β, 5'-GGG CTG ACG GCC AGC GAG GGA-3' and 5'-TGG GGA GAA CCG GGT GCC GA-3'; PPARγ, 5'-GCG GAG ATC TCC AGT GAT ATC-3' and 5'-TCA GCG ACT GGG ACT TTT CT-3'; BSP, 5'-CGC CTA CTT TTA TCC TCC TCT G-3' and 5'-CTG ACC CTC GTA GCC TTC ATA G-3'; OCN, 5'-AGG ACC CTC TCT CTG CTC AC-3'and 5'-AAC GGT GGT GCC ATA GAT GC-3'; Runx2, 5'-CTT CAT TCG CCT CAC AAA C-3' and 5'-CAC GTC GCT CAT CTT GCC GG-3'; L32, 5'-CAT GGC TGC CCT TCG GCC TC-3' and 5'-CAT TCT CTT CGC TGC GTA GCC-3'. The primers for C/EBPα,β,δ, and adipsin were as follows: C/EBPα, 5'-GAA TCT CCT AGT CCT GGC TC-3' and 5'-GAT GAG AAC AGC AAC GAG TAC-3 [40]; C/EBPβ, 5'-GCC ACG GAC ACC TTC GAG G-3' and 5'-CGG CTC CGC CTT GAG CTG-3' [40]; C/EBPδ, 5'-GCG GAT CCG AGG TGA CAG CCC AAC TTG-3' and 5'-GGA ATT CGG TCG TTC GGA GTC TCT AAG-3' [41]; adipsin, 5'-TGT ACT TCG TGG CTC TGG TG-3' and 5'-ATC CGG TAG GAT GAC ACT CG-3' [42]. Real-time PCR was carried out by using the LightCycler system (SYBR Green 1; Roche Diagnostics, Indianapolis, IN) according to manufacturer's instructions. L32 was used as internal control. For semiquantitative assessment of expression levels, each PCR reaction was done over an increasing series of cycles from 17 to 45 cycles and PCR products were size fractionated on 1% ethidium bromide/agarose gels. A representative gel is shown in which the bands are visualized from cycle number within the exponential phase of amplification as determined by densitometric analysis of amplimers (Image Quant software, MD Apps) (see for example, [43]).

### Staining

For lipid-containing adipocyte detection, cells were fixed in 10% neutral buffered formalin and stained with oil red O solution [11]. To detect osteoblasts, cells were incubated with either single or double-stained for ALP and mineral (2.5% silver nitrate (von Kossa)) as described [39]. For single colony analyses, colonies were double-stained with ALP and oil red O.

### Statistics

Experiments were repeated on a minimum of three independent cell isolates. In some cases, as specified in the figure legends, a representative experiment is shown in which data points are the mean ± SD of triplicate samples. Statistical significance was computed by ANOVA and Dunnet's t-test and set at the level of *p *< 0.05 for high density cultures. For low density cultures, at least three independent experiments were done; data were analyzed by Fisher's exact test and significance was set at *p *< 0.05.

## Authors' contributions

KO, YY and JEA designed and coordinated the work, helped with data interpretation and wrote the manuscript. KO, YY and TH conducted most of the experimental work. NM and KT provided critical comments on the manuscript. All authors read and approved the final version of the manuscript.
